# A boon to aged society: Early diagnosis of Alzheimer's disease–An opinion

**DOI:** 10.3389/fpubh.2022.1076472

**Published:** 2022-12-01

**Authors:** Ambily Francis, Immanuel Alex Pandian, J. Anitha

**Affiliations:** ^1^Department of Electronics and Communication Engineering, Karunya Institute of Technology and Sciences, Coimbatore, India; ^2^Department of Electronics and Communication Engineering, Sahrdaya College of Engineering and Technology, Kodakara, India; ^3^Department of Computer Science and Engineering, Karunya Institute of Technology and Sciences, Coimbatore, India

**Keywords:** deep learning, pre-trained network, mild cognitive impairment (MCI), features, cognitively normal (CN), Alzheimer's disease

## Introduction

**Figure 1:**
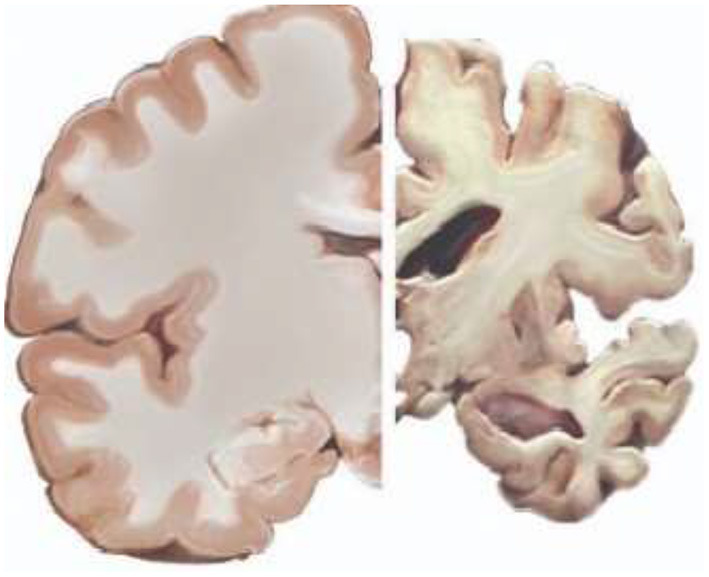
Healthy brain **(Left)** and Alzheimer's affected brain (Right)^*^.

Alzheimer's disease is a highly terrible condition for both the victims and their loved ones to endure. It is a degenerative neurological condition that steadily worsens until the patient is no longer able to do daily tasks. Even though there is no complete cure for the illness, several medications can slow its progression. After the age of 60, every person's brain, notably the hippocampus, begins to shrink (1, 2). But, for some unknown reasons, the shrinking rate will be higher for some people. In this context, the four cognitive stages of the human brain are classified as cognitively normal (CN), mild cognitive impairment convertible (MCIc), mild cognitive impairment non-convertible (MCInc), and Alzheimer's disease (AD) (3, 4). The stages are differentiated in terms of the shrinkage rate of the hippocampus. The hippocampus frequently shrinks as people age. Alzheimer's disease is clearly shown by the hippocampus's unusually rapid rate of atrophy. Cerebrospinal fluid (CSF) will occupy the gray matter area as the hippocampus shrinks. As a result, gray matter falls short of the level needed to maintain proper function. Therefore, the increasing CSF volume is yet another sign of Alzheimer's disease. The healthy brain and Alzheimer's disease affected brain are given in Figure 1 (^*^Credit: Adapted from illustration by Stacy Jannis/Alzheimer's Association, Link: https://www.alz.org/alzheimers-dementia/what-is-alzheimers/brain_tour/credits). The main determinants for classifying various stages of cognition are shrinkage of the hippocampus, growth of the brain's ventricles, and the change from gray matter to CSF (5, 6). The normal aging stage that will not lead to AD is known as mild cognitive impairment non-convertible stage. In this stage memory problems due to normal aging can be identified. However, Alzheimer's disease will never develop from this stage. The early stage of Alzheimer's disease with a significant rate of brain shrinking is known as the mild cognitive impairment convertible stage. The patients with mild cognitive impairment convertible stage will become Alzheimer's patients within years. In the field of early detection of AD, many algorithms address the classification of AD and CN. However, if the system accurately distinguishes between MCIc and MCInc, early detection of AD can be asserted. So the classification between MCIc and MCInc deserves more attention in this field.

## Clinical practices of early diagnosis of Alzheimer's disease

To better comprehend the aberrant state of the human brain, doctors are running clinical trials. Clinical diagnoses are made using anecdotal evidence. The most often used technique for making a clinical diagnosis is the mini-mental state examination. Patients who are undergoing a mini-mental state assessment fill out a questionnaire designed to reflect any brain disorders, such as Alzheimer's disease. For the accurate diagnosis of disease, many conversations with clinicians are necessary.

For the screening, diagnosis, and subsequent management of Alzheimer's disease patients, doctors must be able to quickly and accurately identify the symptoms and pathology of the disease that are associated with Alzheimer's disease. Additionally, it enables patients and others who are caring for them to make necessary lifestyle modifications that may prolong the maintenance of their quality of life. Unfortunately, identifying Alzheimer's disease in its early stages in clinical practice can be difficult and is hampered by several obstacles, including time restrictions on clinicians, difficulty accurately diagnosing Alzheimer's pathology, and the fact that patients and healthcare professionals frequently write off symptoms as being a normal part of aging (7).

## Computer based early diagnosis of Alzheimer's disease

Clinical exams and MRI scan reports may not be able to detect the early stage of AD in patients. If the early illness indications are discovered before the victim notices any symptoms, early detection of Alzheimer's disease gives a great possibility of prompt treatment. The researchers should integrate and evaluate the necessary data for the crucial improvement of medical diagnostic systems. Numerous contributions by researchers addressed the challenges related to the early detection of AD. In recent studies, MRI images are evaluated and processed to find signs of Alzheimer's disease in its early stages (8, 9). Doctors can use the successfully developed system as a clear channel of communication to identify AD in its early stages.

**Figure 2:**
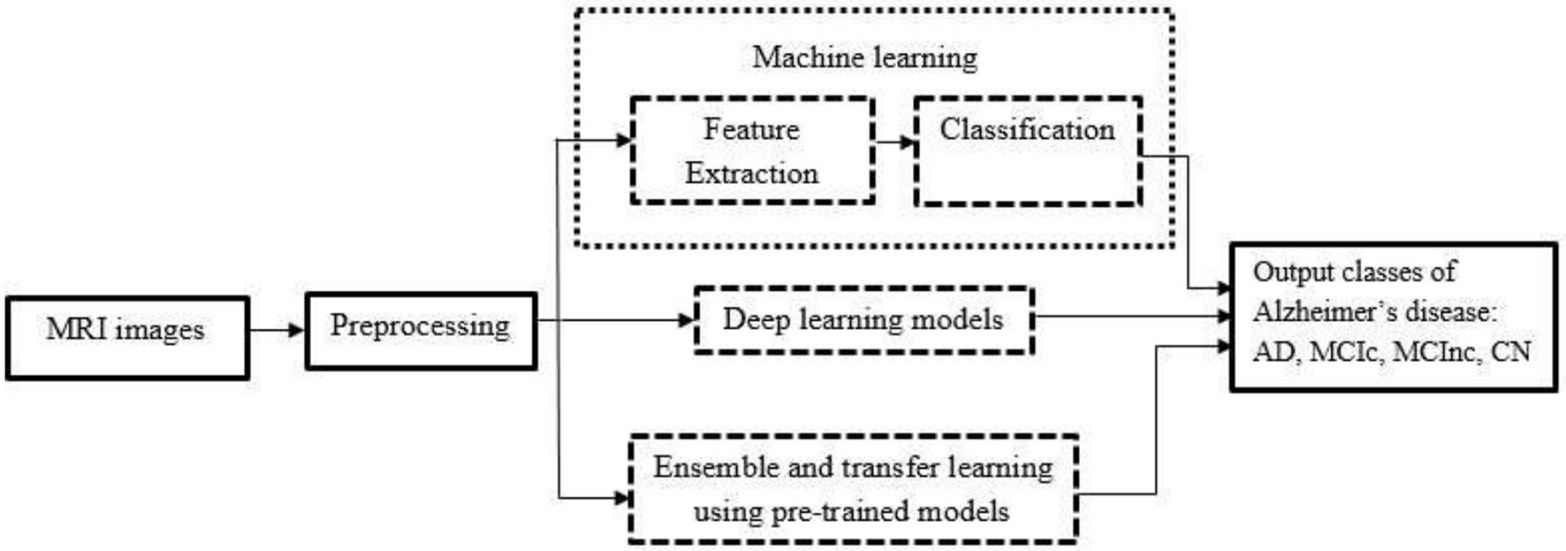
Basic working flow of computer-based methods.

Machine learning techniques, both supervised and unsupervised, are applied to the analysis of medical images. Using supervised learning algorithms, labeled data is categorized using domain knowledge from an expert and pertinent feature data. The unsupervised learning algorithms work on unlabeled data. The supervised and unsupervised deep learning models are capable of automatically creating feature extractors and extracting discriminative features from train data. The rest of this section is structured as follows. Early diagnosis of AD using conventional feature extraction methods are deliberated in section Early diagnosis based on conventional feature extraction methods. Section Early diagnosis of Alzheimer's disease based on deep learning techniques describes related works on early diagnosis of AD using deep learning techniques. In section Early diagnosis of Alzheimer's disease based on pre-trained models, related works for early diagnosis of AD based on pre-trained models are discussed. The basic working flow of three computer-based methods for the early diagnosis of Alzheimer's disease are portrayed in Figure 2.

### Early diagnosis based on conventional feature extraction methods

Feature descriptors play an important role in medical image analysis. Medical image processing applications that explicitly use feature descriptors include disease diagnosis (10, 11), medical joint photographic experts group image steganography (12), object recognition, and segmentation. The two types of feature descriptors are global feature descriptors and local feature descriptors. Global features are those that apply to the entire image. Local features are provided by patches of an image. For any application involving the analysis of medical images using conventional feature extraction methods, global features are insufficient as they cannot provide the spatial information of images. For early Alzheimer's disease identification, many works suggested novel local feature descriptors.

Texture features are highly important low-level features that give significant details about specific regions of medical images. The pixel intensities of image patches are used to obtain the texture features. Numerous texture descriptors, including the local binary pattern, the scale-invariant feature transform, and the speed- up robust features, are used to interpret MRI images. The well-known descriptor, local binary pattern (LBP) is proposed for describing texture qualities (13). A high classification accuracy is achieved at the expense of a significant calculation time by using texture features taken from an elliptical neighborhood (14). Two-dimensional and three-dimensional advanced local binary patterns are combined to obtain accurate multi-class Alzheimer's disease prediction (15). The computational time taken for the processing of high-dimension features is the major limitation of this method. The textural features are identified by considering the voxel neighbors of MRI image (16–19).

Scale-invariant feature transform (SIFT) is another interesting local feature descriptor. The scale invariance and rotation invariance are the important characteristics of SIFT. Both the frequency domain and the spatial domain are utilized for extracting SIFT features. SIFT is effectively used for the early detection of AD (20–23). According to the discussions in the aforementioned articles, the benefit is that SIFT did not alter with variations in illumination. However, the gradients of each voxel along the path must be calculated. Hence the computation must be regarded as intensive.

The Hessian matrix serves as the foundation for the Speeded Up Robust Features (SURF) which shares many characteristics with SIFT (24). SURF performs admirably in computer vision applications and has been researched for use in medical image applications (5, 25, 26). The primary feature of SURF is the enhanced speed that the integral filter produces. As previously stated, one key sign of AD is the increasing CSF volume in the hippocampus. CSF volume computation is used to carry out early identification of AD (5). The CSF volume computation is done based on the number of CSF voxels in the hippocampal area. Circular harmonic functions (CHF) offers great image description independent of scale, position, and illumination for the early diagnosis of AD (27, 28).

After preprocessing, MRI images are sampled with nine volumes of interest in brain regions related to AD. Intensity and texture features are extracted from the interest of volume. The support vector machine classifier trains on the features. Non-linear registration systems can be made better even when the method offers tolerable accuracy (29). Clinical and texture characteristics are integrated to identify the mild cognitive impairment convertible stage (MCIc). The key benefit is that MCI (Mild cognitive impairment) and AD have been classified using the entire brain's MRI texture with binary logistic regression (30). A feature selection technique that makes use of a multivariate general linear model is proposed for the MCIc vs. CN classification. The modest intensity fluctuations from CN to MCIc are produced with the use of a general linear model. Additionally, multivariate adaptive regression splines are utilized as a classifier (31).

The orientation surrounding each voxel of eight local regions of MRI image including white matter and gray matter is calculated using a 3D local directional pattern. The algorithm is less susceptible to illumination and noise (11). The features obtained from many modalities are combined using multi- kernel SVM. In this method hyper graph-based regularization is used for AD vs. MCI classification. The method provides superior classification accuracy than existing multi-modality strategies, according to the results. The algorithm's primary flaw is that all hyperedge weights are set to 1 without considering various hyperedges (32). It is possible to identify Alzheimer's disease more quickly by analyzing data sets of medical records using the machine learning algorithms such as Logistic Regression, Decision Tree, Random Forest, Naive Bayes and variants of support vector machine to identify Alzheimer's infection (33).

### Early diagnosis of Alzheimer's disease based on deep learning techniques

In recent years, deep learning methods have been prominent in many medical sectors. For efficient and precise categorization, deep learning techniques will abstract important features from the images. The methods aid medical professionals in early disease diagnosis. These methods enhance researchers' abilities to engage in studies that examine medical image approaches for disease diagnosis. Shortly, deep learning may replace traditional medical diagnostic techniques because of its learning capabilities and cost-effectiveness. A sizable number of convolutional neural network (CNN) models are combined to successfully anticipate various stages of AD. The classification performance is good as a result of the integration of Bagging, Boosting, and Random Forest algorithms (34). The review (35) listed and examined the most recent studies in the area of early Alzheimer's disease diagnosis using deep learning algorithms. Using 2D MRI slices, the work (36) proposes a CNN-based method for extracting discriminatory characteristics from structural MRI, intending to categorize Alzheimer's disease and healthy people.

The algorithm (37) uses functional MRI images in addition to medical data like age, gender, and genetic information. Using stacked autoencoders and functional MRI time-series data or correlation coefficient data, the deep neural network is trained. The research (38) reviews a number of deep learning based algorithms used in the early identification of Alzheimer's disease. The approach also offers a flexible foundation for repeatable testing. The strengths of hessian matrix and local binary pattern are utilized with the use of convolutional neural network (4). The algorithm provides best classification accuracy for AD vs. CN. But the Classification accuracy of MCIc vs. MCInc can be improved.

### Early diagnosis of Alzheimer's disease based on pre-trained models

Transfer learning and ensemble learning are typically expressed in computer vision by employing pre-trained models. The innovative approaches for dealing with the issue of training data and test data having different distributions include transfer learning and ensemble learning. Pre-trained models can be used as the foundation for new models for the early diagnosis of AD. Using a neural network model, transfer learning is utilized to improve the precision and computing efficiency of image categorization. In transfer learning models, pre-trained neural networks are used as feature extractors, and the output of the pre-trained network is given to a new classification layer that is trained on data particular to the task. By integrating many previously trained models with new classification layers, ensemble transfer learning models (39, 40) are utilized to increase performance at the cost of increased model complexity. Without much computational complexity, the squeeze and excitation network affects the channel attention process by strengthening and weakening each feature channel. The algorithm (41) illustrates that non-biomedical pre-trained models, such as ResNet, learn cross-domain characteristics that enable the model to extract critical low-level properties from MRI scans to improve classification accuracy. The suggested method guarantees effective data augmentation before learning. The augmentation helps to regularize the model.

A 3D multi-channel feature maps based on Voxception-Resnet is created for the classification of AD and CN (42). Data augmentation is done before feature map creation. The dataset used in this algorithm is diffusion MRI images. The work (43) employs the VGG-16 pre-trained model, a non-biomedical model, to learn cross-domain characteristics and boost accuracy. The method achieves exceptional three-class classification accuracy and offers a mathematical model based on VGG-16 transfer learning. The method (44) uses 3D DenseNet to learn both hippocampus segmentation and classification features that based on deep CNN. Alzheimer's checking web application is proposed based on transfer learning of pre-trained models such as VGG19 (45).

## Discussions

As discussed in the previous sections, AD develops gradually that takes years for symptoms to physically manifest in a patient, thus clinical approaches for an early diagnosis of the disease are insufficient. Physical symptoms of AD in its early stages will resemble those of a typical aged person in many ways. Clinical approaches are not very reliable for separating mild cognitive impairment into convertible and non-convertible stages in the context of early diagnosis.Early diagnosis is made using MRI scans using image processing techniques. The early diagnosis of AD utilizing MRI and Positron Emission Tomography (PET) image modalities is made possible by numerous algorithms (46, 47). Algorithms that need manual feature extraction to take a lot of time and have high computational complexity. Additionally, hardware implementation of such a system necessitates an extremely complicated system. Also, these kinds of systems only provide very poor MCIc vs. MCInc classification accuracy. The distinction between phases MCIc and MCInc is very difficult because of very slight voxel changes. Thus, the difference between MCIc and MCInc images may be described by a high-end feature description.

For the early diagnosis of AD, deep learning algorithms play a significant role. Numerous algorithms are developed for deep learning-based early diagnosis. Reduced computational complexity, less calculation time, low dimension features and good differentiation capability between MCIc vs. MCInc should be the goal of the computer-based diagnostic system. The pre-trained models successfully adapt the MRI images to predict the early phase of AD (48). The high-dimension features of pre-trained models cause complexity in the physical implementations later. But with pre-trained models, MCIc vs. MCInc classification accuracy is fairly good at the cost of computational complexity, processing time, and high dimension features.

Many investigations tried to classify AD vs. CN, and AD vs. CN vs. MCI (not specifically MCIc or MCInc). But only less focus is given to the classification of MCI vs. MCInc. Even though early Alzheimer's disease detection has been the subject of countless research, it is still challenging to identify the specific traits that can detect the disease in its earliest stages (49). The most salient classification for the early identification of AD is MCIc vs. MCInc. The objective of the upcoming efforts is to increase the classification precision of MCIc vs. MCInc. Due to extremely slight voxel changes, the differentiation between phases of MCIc and MCInc is exceedingly laborious. According to the published researches, MCIc vs. MCInc categorization accuracy ranges from 55 to 75% (38, 50, 51). Further study is necessary due to the low categorization accuracy for MCIc vs. MCInc. Unique learning strategies that discriminate between mild cognitive impairment convertible and nonconvertible stages will accelerate the classification accuracy of MCIc vs. MCInc. The reliable, fully automated system for the early diagnosis of AD can be a boon to the aged society in the near future.

## Author contributions

AF, IP, and JA contributed to the study conception and design, literature review, interpretation, and manuscript preparation. All authors contributed to the article and approved the submitted version.

## Conflict of interest

The authors declare that the research was conducted in the absence of any commercial or financial relationships that could be construed as a potential conflict of interest.

## Publisher's note

All claims expressed in this article are solely those of the authors and do not necessarily represent those of their affiliated organizations, or those of the publisher, the editors and the reviewers. Any product that may be evaluated in this article, or claim that may be made by its manufacturer, is not guaranteed or endorsed by the publisher.
